# Allelic association studies of genome wide association data can reveal errors in marker position assignments

**DOI:** 10.1186/1471-2156-8-30

**Published:** 2007-06-08

**Authors:** David Curtis

**Affiliations:** 1 Academic Centre for Psychiatry, Queen Mary's School of Medicine and Dentistry, London E1 1BB, UK

## Abstract

**Background:**

Genome wide association (GWA) studies provide the opportunity to develop new kinds of analysis. Analysing pairs of markers from separate regions might lead to the detection of allelic association which might indicate an interaction between nearby genes.

**Methods:**

396,591 markers typed in 541 subjects were studied. 7.8*10^10 ^pairs of markers were screened and those showing initial evidence for allelic association were subjected to more thorough investigation along with 10 flanking markers on either side.

**Results:**

No evidence was detected for interaction. However 6 markers appeared to have an incorrect map position according to NCBI Build 35. One of these was corrected in Build 36 and 2 were dropped. The remaining 3 were left with map positions inconsistent with their allelic association relationships.

**Discussion:**

Although no interaction effects were detected the method was successful in identifying markers with probably incorrect map positions.

**Conclusion:**

The study of allelic association can supplement other methods for assigning markers to particular map positions. Analyses of this type may usefully be applied to data from future GWA studies.

## Background

A number of genome wide association (GWA) studies are now being completed and some results are becoming publicly available. These results offer the possibility to carry out a number of new types of analysis. For example, it may well be that relatively common polymorphisms at unlinked loci could have an interactive effect on the phenotype and that this effect might be detectable as a distortion of the observed joint genotype frequencies. If two genes carry out a similar function but only one copy of either is needed for survival then a population might sustain relatively common loss-offunction mutations. Only if these occurred in both copies of both genes would an organism not be viable. Such double-recessive lethal mutations might exert a relatively small effect on selection and might only cause a small deviation from Hardy-Weinberg equilibrium (HWE) at each locus separately. However were the genotypes of markers close to both loci to be considered jointly it might become apparent that there was a very marked reduction in the number of subjects being homozygous at both loci for alleles associated with the mutations. Effects such as this could be detectable through finding allelic association between pairs of markers far apart on the genome. Such an investigation would fall into the general category of case-only tests for interaction [1].

This report presents the results obtained by studying publicly available data from a GWA in order to search for distortions in joint genotype frequencies between pairs of markers. It was expected that such distortions might lead to the identification of pairs of genes have some functional similarities and hence possibly harbouring variations with an epistatic effect on survival.

## Results

Pairs of markers genotyped in the course of a GWA were identified as showing allelic association with each other in spite of being separated by a very large genetic distance. There were 84 pairs of groups selected for analysis following an initial screening procedure to identify long range allelic association, each group containing 21 contiguous markers. Analysis of all pairs of markers between groups failed to identify any pair meeting the pre-defined criterion for indicating probable epistatic interaction affecting survival. That is, in each case only a single marker from one group demonstrated allelic association with one or more markers in the other group.

For most of the pairs of groups only very weak allelic association was detectable between and within groups and hence they were discarded as not demonstrating any readily interpretable effect.

A number of pairs of groups seemed to demonstrate evidence for a marker to be incorrectly assigned with respect to its map position. Although there were 30 pairs of groups in which this occurred, closer inspection revealed that this was due to effects of 6 individual markers. The reason that more groups than this were identified is that the algorithm for identifying overlapping sets of markers was not perfect. Each of these 6 markers demonstrated very strong evidence for allelic association with markers in a distant region while demonstrating no allelic association at all with any of the markers which were supposed to be flanking it. These 6 markers are listed in Table 1, which shows the position according to NCBI Build 35, the position according to NCBI Build 36 and the approximate position according to the markers with which strong allelic association is detected. It can be seen that for one of the markers, rs4144700, the position has been changed between builds and the newly assigned position accords well with the results of LD analysis. Two of the markers, rs1050301 and rs3189745, are not included in the newer build while for the other three, rs2016844, rs2037375 and rs8812, the assigned positions remain in conflict with the results of allelic association analysis. Although not formally quantified, it was striking that in general the flanking markers demonstrate some allelic association with one or more other markers in the same group. Hence, the apparently wrongly assigned markers were exceptional in not demonstrating any allelic association with markers which were supposed to lie nearby.

**Table 1:** Assignment of marker positions according to published databases and to allelic association results

**Marker name**	**Assigned position (chromosome:bp)**		
	**NCBI Build 35**	**NCBI Build 36 (from UCSC browser)**	**By allelic association relationships (approximate)**

rs2016844	chr11:8895510	chr11:8895510	chr3:156341383
rs1050301	chr21:44922061	(not found)	chr2:86152633
rs2037375	chr12:88585377	chr12:88607040	chr1:33844112
rs4144700	chr11:88729775	chr11:49327410	chr11:49129810
rs3189745	chr2:76397507	(not found)	chr14:20010355
rs8812	chr19:23101782	chr19:23101782	chr14:34118096

## Discussion

Although the method has been unsuccessful in detecting pairs of genes interacting epistatically to affect survival it has highlighted a number of markers for which the assigned positions appeared to be incorrect. The fact that one of these markers has already had its position corrected and that two more have been omitted provides some support for the validity of this approach.

## Conclusion

It seems that allelic association studies could be used to supplement other methods for assigning markers to particular map positions. Only the markers which showed the strongest evidence for an incorrect assignment in the screening analysis were selected and it may well be that there are others which could be picked up by using a similar approach. Systematic testing of all pairs of markers could be carried out. At minimum, checks could be carried out on those markers which show no allelic association with those which are supposed to surround them. GWA datasets involving large numbers of subjects will shortly be becoming available and these will offer the opportunity to carry out more detailed and sensitive studies of this nature.

## Methods

This study used data from the SNP Database at the NINDS Human Genetics   Resource Center DNA and Cell Line Repository [3]. The dataset consisted of 541 subjects, both cases and controls, genotyped for a GWA of Parkinson's disease using the Illumina Infinium I and Infinium II assays [2]. The original genotyping was performed in the laboratory of Drs. Singleton and Hardy (NIA, LNG), Bethesda, MD USA. Also downloaded was information concerning the markers used, including their positions as   reported from NCBI Build 35 [4].

All pairs of 396,591 autosomal markers were assessed for initial evidence of allelic association except for those within 1,000 markers of each other. Since this required carrying out in the order or 7.8*10^10 ^analyses, a rapid screening procedure was used to identify pairs which might potentially be of interest. This consisted of selecting pairs for which the observed number of subjects having one of the four joint homozygote genotypes (AA-AA, AA-BB, BB-AA or BB-BB) differed from that which would be expected from the separate genotype frequencies by a particular threshold value of *z *based on a binomial expectation, where *z *= (*O*-*E*)/√*E *with *O *being the observed count and *E *the expected count. This method was implemented in a custom C program with optimisations aimed at performing the large number of comparisons required in a reasonable length of time. A threshold value of *z *> 6 was used to identify pairs worthy of further investigation. Such a value has an asymptotic significance value of 10^-9 ^so one would not expect a very large number of such results to occur by chance even taking into consideration the large number of pairs tested.

Pairs identified by the screening procedure were subjected to more detailed analysis. Each member of the pair was included along with 10 markers on either side of it to form two groups of 21 markers each. Then R^2 ^was calculated between all pairs of markers within each group and all pairs of markers between the groups using the *LDPAIRS *program [5]. Sometimes a marker would show initial evidence for allelic association with several other markers which would be close together and which would end up in the same group. An attempt was made to identify this situation and then to use each group only once rather than repeating analyses for every pair highlighted by the screening procedure. However limitations of computer memory made it impossible identify all overlaps and so some sets of analyses were carried out using similar groups of markers. It is possible that a more sophisticated algorithm could have been devised for grouping markers but the procedure as described was not too prohibitive in terms of time.

If the results of the screening procedure were due to a joint effect of interacting mutations one might expect additional pairs of markers between the two regions to show some allelic association, perhaps at a lesser magnitude. In order to assign a pair of markers as probably indicating an interaction between two genes evidence was sought for allelic association (R^2 ^> 0.1) between a different pair of markers, one from each group, or for both members of the original pair showing evidence for allelic association (R^2 ^> 0.1) with at least one additional marker each. Thus there would be at least two markers in each group showing evidence for allelic association with a marker in the other group.

It soon emerged that some markers might be showing allelic association because the assigned map positions were incorrect. A marker was identified as being probably incorrectly assigned if it showed definite evidence of allelic association with more than one marker in the other region and no evidence for allelic association with markers from the group it was supposed to be assigned to. The thresholds used to indicate probable incorrect assignment were that there would be a value of R^2 ^greater than 0.4 for at least one additional marker from the other group apart from the one which had been highlighted in the initial screening and that there would be no value of R^2 ^greater than 0.1 for any of the other 20 markers which were supposed to flank it.

Once markers were identified as being possibly incorrectly assigned the position according to NCBI build 36 was looked up in the UCSC browser [6].
